# Cheating the Locals: Invasive Mussels Steal and Benefit from the Cooling Effect of Indigenous Mussels

**DOI:** 10.1371/journal.pone.0152556

**Published:** 2016-03-31

**Authors:** Justin A. Lathlean, Laurent Seuront, Christopher D. McQuaid, Terence P. T. Ng, Gerardo I. Zardi, Katy R. Nicastro

**Affiliations:** 1 Department of Zoology and Entomology, Rhodes University, Grahamstown 6140, South Africa; 2 Centre National de la Recherche Scientifique, CNRS UMR 8187 LOG, 28 avenue Foch, BP 80, 62930 Wimereux, France; 3 The Swire Institute of Marine Science and School of Biological Sciences, The University of Hong Kong, Pokfulam Road, Hong Kong SAR, China; 4 CCMAR—Centro de Ciencias do Mar, CIMAR Laboratório Associado, Universidade do Algarve, Campus de Gambelas, 8005–139 Faro, Portugal; College of Charleston, UNITED STATES

## Abstract

The indigenous South African mussel *Perna perna* gapes during periods of aerial exposure to maintain aerobic respiration. This behaviour has no effect on the body temperatures of isolated individuals, but when surrounded by conspecifics, beneficial cooling effects of gaping emerge. It is uncertain, however, whether the presence of the invasive mussel *Mytilus galloprovincialis* limits the ability of *P*. *perna* for collective thermoregulation. We investigated whether varying densities of *P*. *perna* and *M*. *galloprovincialis* influences the thermal properties of both natural and artificial mussel beds during periods of emersion. Using infrared thermography, body temperatures of *P*. *perna* within mixed artificial beds were shown to increase faster and reach higher temperatures than individuals in conspecific beds, indicating that the presence of *M*. *galloprovincialis* limits the group cooling effects of gaping. In contrast, body temperatures of *M*. *galloprovincialis* within mixed artificial mussel beds increased slower and exhibited lower temperatures than for individuals in beds comprised entirely of *M*. *galloprovincialis*. Interestingly, differences in bed temperatures and heating rates were largely dependent on the size of mussels, with beds comprised of larger individuals experiencing less thermal stress irrespective of species composition. The small-scale patterns of thermal stress detected within manipulated beds were not observed within naturally occurring mixed mussel beds. We propose that small-scale differences in topography, size-structure, mussel bed size and the presence of organisms encrusting the mussel shells mask the effects of gaping behaviour within natural mussel beds. Nevertheless, the results from our manipulative experiment indicate that the invasive species *M*. *galloprovincialis* steals thermal properties as well as resources from the indigenous mussel *P*. *perna*. This may have significant implications for predicting how the co-existence of these two species may change as global temperatures continue to rise.

## Introduction

Thermoregulatory behaviour often plays a crucial role in improving the performance of ectotherms and maintaining viable populations in harsh and variable environments [1,2]. Behavioural thermoregulation is the primary method of thermal homeostasis for the many ectotherms that are incapable of thermogenesis [1], but to date, most of our knowledge of such behaviour comes from terrestrial reptiles, amphibians and insects [3,4] and to a lesser extent intertidal invertebrates [5–8]. These studies report diverse types of thermoregulatory behaviours, including posturing in lizards and gastropods [9–11], aggregation in snakes [12], marine snails [6], and mussels [13], habitat engineering in termites [14], active microhabitat selection in amphibians [15] and intertidal gastropods [5,6,16] and regional heterothermy in seastars [17]. In most cases, thermoregulatory behaviour has only been considered at the individual level or in the context of intraspecific interactions. Noticeably, much less is known about how the thermoregulatory behaviour of one species influences the thermal properties of another.

Two intertidal mussels with different responses to air exposure dominate rocky intertidal communities on the south coast of South Africa: the indigenous mussel *Perna perna* and the invasive mussel *Mytilus galloprovincialis*. The two co-exist on shores with partial habitat segregation, *M*. *galloprovincialis* generally occurring higher on the shore, but with a region of overlap where the two occur in mixed beds [18]. During emersion, *P*. *perna* exhibits periodic closure and opening of the shell (gaping); this behaviour allows the maintenance of aerobic respiration, but increases levels of water loss and the risk of desiccation due to both evaporation and expulsion of water during valve closure. In contrast, *M*. *galloprovincialis* keeps its valves closed when exposed to air. This behaviour reduces the risk of desiccation, but decreases the efficiency of anaerobic respiration [19]. These different responses to aerial exposure, along with different byssal attachment strengths, are thought to contribute to the different vertical distributions of the two species [18, 20–22].

Recent laboratory experiments reveal that the gaping behaviour of *P*. *perna* is ineffective in reducing body temperatures of solitary mussels, but that when individuals are surrounded by conspecifics, evaporative cooling effects emerge for the group as a whole [23]. Consequently, increasing densities of *M*. *galloprovincialis* within *P*. *perna* beds may reduce the ability of individual *P*. *perna* to thermoregulate, whilst allowing *M*. *galloprovincialis* to ‘steal’ beneficial thermal properties from *P*. *perna*. This hypothesis is consistent with observations that, during periods of severe heat-stress, mortality rates of *P*. *perna* surrounded by non-gaping *M*. *galloprovincialis* in the field were higher than those of *P*. *perna* surrounded by conspecifics [23].

We investigated the thermal relationship between *M*. *galloprovincialis* and *P*. *perna* by testing the effect of varying densities of the two species on the thermal properties of both natural and artificial mussel beds during periods of aerial heat-stress of various intensities. Increasing densities of non-gaping *M*. *galloprovincialis* within the midshore region and in artificial mussel beds were expected to limit the ability of *P*. *perna* to thermoregulate and thus increase the amount of heat-stress experienced by both species of mussels. Conversely, increasing densities of gaping *P*. *perna* were expected to lower the thermal stress experienced by neighbouring *M*. *galloprovincialis*.

## Materials and Methods

### Study location

**Fig 1:**
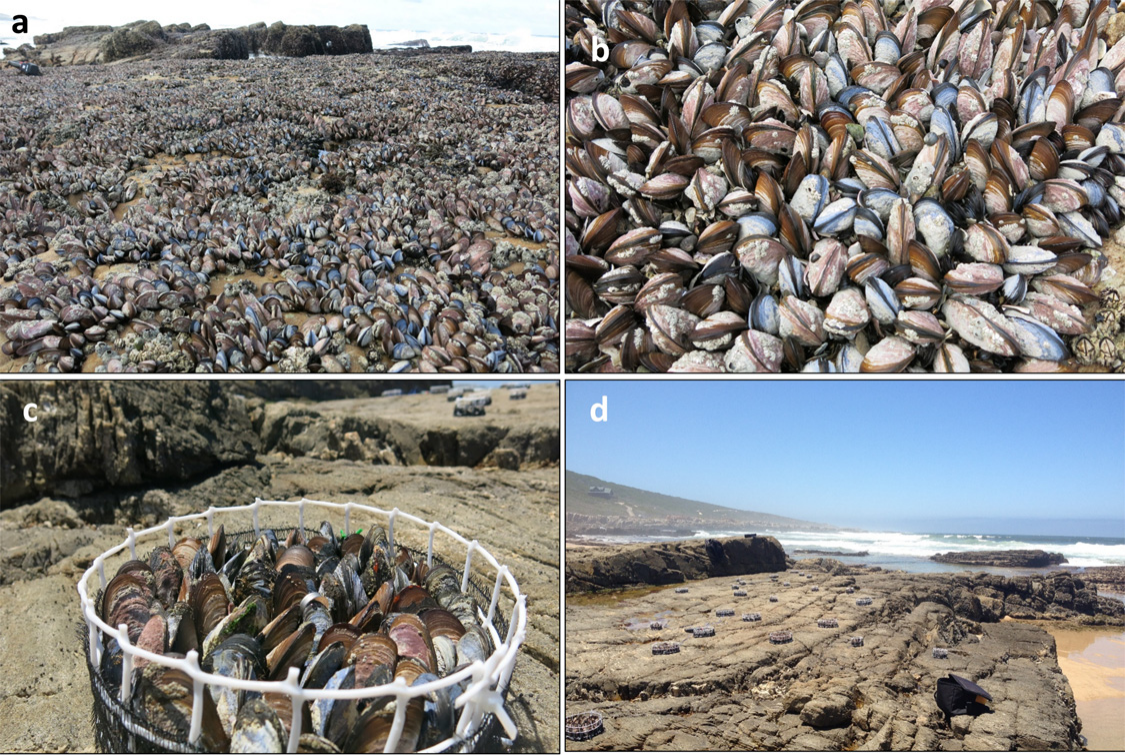
(a-b) Photos of mixed mussel beds within the midshore region at Jongensfontein, South Africa. The invasive mussel *Mytilus galloprovincialis* is easily distinguished from the indigenous mussel *Perna perna* by its blue colouration. (c-d) Experimental manipulations of mussel beds: artificial mussels beds were created by filling circular cages (20 cm diameter) with the desired size and density of the two mussel species (c). These were then placed high on the shore during a summer low-tide to simulate an extreme heat-event (d).

The study was undertaken on a moderately exposed rocky intertidal shore at Jongensfontein (34°25’12.26”S, 21°21’28.27”E) on the south coast of South Africa, where both species are abundant in the mid-shore region and display similar size class distributions (S1 Fig). The site has moderate topographic variability, with most of the shore comprising horizontal surfaces (<10° inclination), with few crevices, pits, boulders or rock pools (Fig 1A), all of which can influence small-scale temperature variability [24]. The rocky substratum is comprised of granite and experiences a semi-diurnal tidal cycle with a tidal range of 1.5 to 2 metres. Field permits were acquired from the South African Department of Agriculture, Forestry and Fisheries (DAFF).

### Natural variability

To investigate the effect of the proportions of *P*. *perna* and *M*. *galloprovincialis* on small-scale thermal properties of natural mussel beds, we took infrared (IR) images of 27 to 30 permanent 20 cm × 20 cm plots along a 50 m transect running parallel to the shore within the midshore region during the lowest daytime tidal cycles in the austral summer, between the 20 and 23 January 2015. Plots were haphazardly selected 1–2 m apart in areas with 100% cover of mussels on flat to slightly sloping (<10° inclination) rocky substrata (Fig 1B). High resolution digital images were used to estimate percent cover of *P*. *perna* and *M*. *galloprovincialis*, using the software package Coral Point Count with Excel Extensions (CPC_E_) version 3.6 [25], which overlaid an evenly spaced matrix of 144 points on each image. Infrared images of each plot were taken using a FLUKE Ti25 IR camera twice every day during daytime aerial exposure, i.e. an hour before and after low-tide. Camera settings and specifications were comparable to other intertidal studies that have used IR thermography [26,27] with emissivity values set at 0.95, thermal sensitivities of 0.09°, and accuracies of ±2°C or ±2% of reading, whichever was greater. Maximum daily air temperatures recorded at a nearby (~10 km) coastal weather station ranged from 26.1°C to 31.3°C during the four-day sampling period (South African Weather Service 2015), producing a range of thermal conditions including extreme heat events within the rocky intertidal zone at the field site.

Mean *P*. *perna* and *M*. *galloprovincialis* surface temperatures for each plot were calculated using the single point measurement tool in the FLUKE SmartView 3.7.23 software package. Single pixels within each IR image representing the surface temperatures of 10 randomly selected *P*. *perna* and *M*. *galloprovincialis* individuals were selected and averaged to give a mean surface temperature for the two species within each plot (N.B. different individuals were chosen for each of the eight sampling events). Surface temperatures of intertidal molluscs measured by IR imagery have previously been shown to be highly correlated with internal body temperatures [28]. Within the present study, comparisons between shell surface temperatures of mussels measured by IR imagery and internal body temperatures measured with digital thermocouples (4 Channel Data Logging Thermometer 800024, SPER SCIENTIFIC Ltd.) also revealed highly significant positive correlations when assessed in the field (r^2^ = 0.96, n = 122, p<0.001, y = 0.9722x+1.1239). Mussel shell surface temperature were shown to be a consistent proxy for mussel body temperature over a wide range of temperature values (from 22°C to 40°C). We therefore refer to shell temperatures measured by IR imagery as body temperatures throughout the rest of the manuscript.

### Density manipulations

Artificial mussel beds were used to test experimentally whether *M*. *galloprovincialis* benefits from the gaping behaviour of *P*. *perna* and/or whether *P*. *perna* experiences increased heat-stress when interspersed with *M*. *galloprovincialis* in mixed mussel beds. These beds were constructed by removing individuals from the midshore region, cleaning the outer surface of encrusting organisms and placing them inside open circular cages (20 cm diameter) made of coarse plastic mesh (Fig 1C; adapted from [23]). Cages were filled with either (i) 100% *P*. *perna*, (ii) 100% *M*. *galloprovincialis*, or (iii) 50% *P*. *perna* and 50% *M*. *galloprovincialis* (hereafter referred to as 50:50 mixed) with species interspersed haphazardly. This design was replicated using both large (6 to 7.5 cm shell length) and small individuals (4 to 5.5 cm shell length) to test whether the effect of species composition on thermal properties of mussel beds varies depending on the size of individuals. Once assembled, all cages (n = 3 per treatment due to limited number of large *M*. *galloprovincialis*) were submerged in a large open rock pool for 90 mins before simultaneously being removed at midday and interspersed across a large flat section of rocky substrata high on the shore, where they remained for 120 mins (Fig 1D). This rock pool was chosen because it was large and remained permanently open to the nearshore environment. Thus the water temperature experienced by mussels during this incubation period would have been equivalent to that experienced by submerged mussels on the rocky shore during high tide. Many individuals had reattached themselves to neighbouring mussels using their byssal threads whilst being submerged. This suggests mussels were responding well to being removed and were not physiologically stressed before the beginning of the experiment.

A combination of IR thermography and temperature data loggers was used to measure the temporal and spatial variability in heat-stress amongst treatments. Data loggers (iButton DS1923L) with a thermal resolution of 0.0625°C and an accuracy of 0.5°C were placed on top of each artificial mussel bed to measure changes in bed temperature at a sampling frequency of 2 mins over the course of the experiment. Temperatures recorded by these iButtons were found to be highly correlated with mean body temperatures of several individual mussels within artificial beds and therefore represent good proxies for measuring changes in bed (or habitat) temperatures (J.A. Lathlean, pers. obs). IR images of each artificial bed were taken every 5 mins, starting immediately after the cages were removed from the water, in order to assess both temporal and spatial variability in the body temperatures of multiple individuals within the same bed. Ten randomly selected *P*. *perna* and/or *M*. *galloprovincialis* individuals were selected within the centre of each cage to limit potential edge effects for body temperature measurement using the single point measurement tool in the FLUKE SmartView 3.7.23 software package. These ten measurements were averaged to give mean estimates of body temperatures of the two species within each bed.

### Data analysis

Pearson correlation analysis was used to test whether the percent cover of *P*. *perna* within a plot influenced absolute temperatures in mean body temperatures of *P*. *perna* and *M*. *galloprovincialis* during the eight sampling events. A three-way ANOVA was used to test whether mean body temperatures of mussels differed between (i) species (orthogonal, fixed, 2 levels: *P*. *perna* or *M*. *galloprovincialis*), (ii) sampling dates (orthogonal, fixed, 4 levels: 20^th^, 21^st^, 22^nd^, 23^rd^ January), and (iii) time of day (orthogonal, fixed, 2 levels: before and after low-tide). This was undertaken to assess whether mean body temperature of mussels differed between the eight sampling events due to the prevailing abiotic conditions. For the manipulative experiment, two-way ANOVAs were used to test whether density treatments (orthogonal, fixed, 3 levels: 100% *P*. *perna*, 100% *M*. *galloprovincialis*, 50:50 mixed) and the size of individuals (orthogonal, fixed, 2 levels: large or small) influenced bed or body temperatures of *P*. *perna* and *M*. *galloprovincialis* within artificial mussel beds. These two-way ANOVAs included separate tests for both absolute temperature and rates of change (degrees per min) for each of the response variables, both halfway through and at the end of the experimental period. Data were normally distributed and showed equal variances using the Shapiro-Wilks test for normality and Cochran’s test, respectively.

## Results

### Natural variability

**Fig 2:**
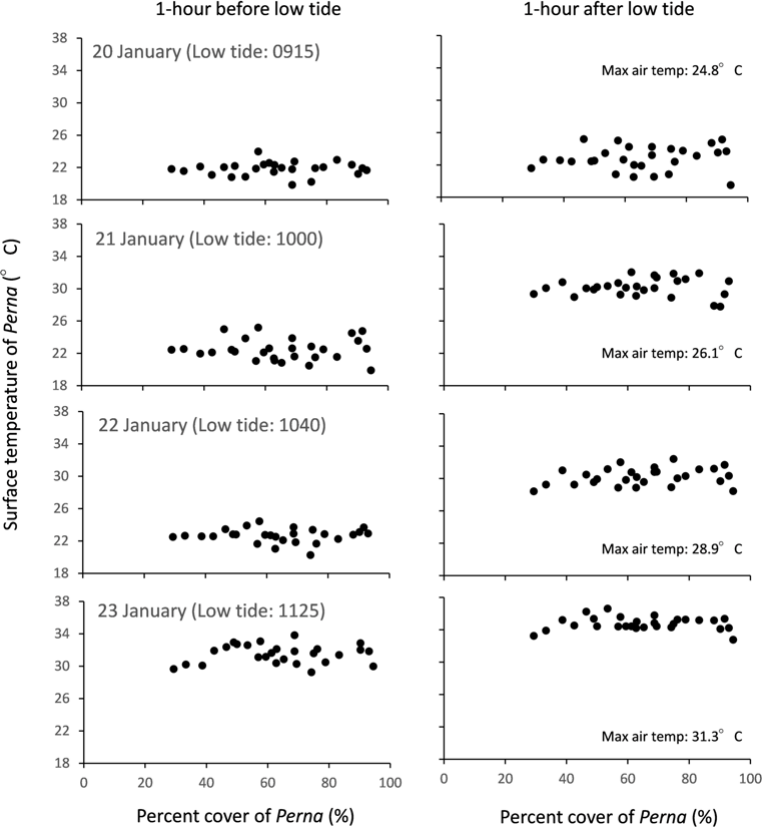
Relationship between percent cover of *P*. *perna* and the mean surface temperatures of *P*. *perna* within naturally occuring midshore mussel beds during four consecutive day-time summer low-tides. Left column represents measurements taken approximately one hour before low-tide; right column represents measurements taken approximately one hour after low tide.

**Fig 3:**
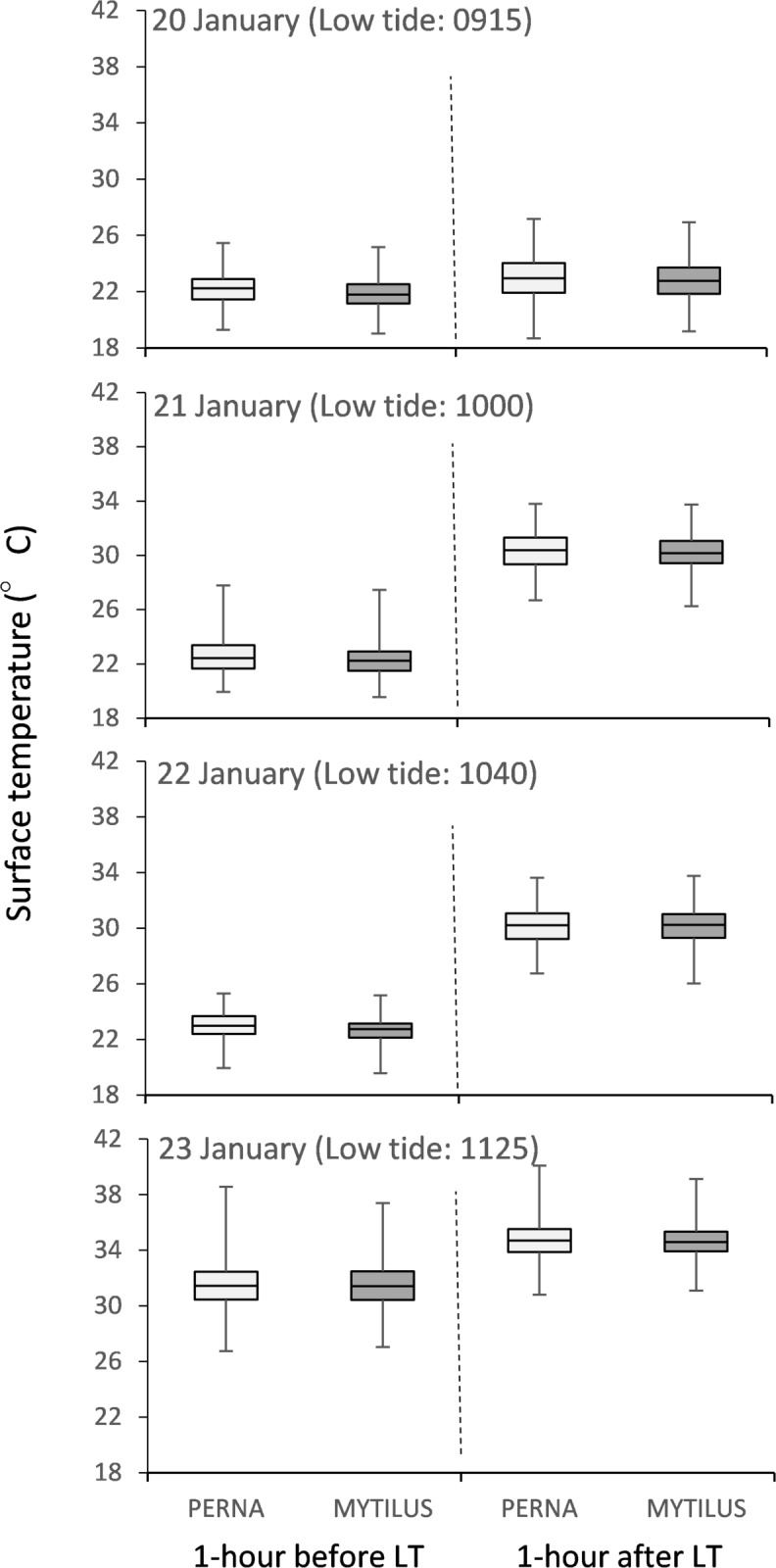
Small-scale temperature variability amongst individual mussels within the midshore region at Jongensfontein one hour before and one hour after low tide (LT) during four consecutive days in January 2015. Box plots represent median, interquartile range, maximum, and minimum. Sample sizes range from 250–290 individuals.

Body temperatures of *P*. *perna* did not vary in response to differences in the percent cover of *P*. *perna*, irrespective of sampling date or whether measurements were taken before or after low-tide (Fig 2, Table 1). In addition, body temperatures of *P*. *perna* were not significantly different from those of *M*. *galloprovincialis* (Fig 3), irrespective of sampling event. Instead, body temperatures of both species appeared to be influenced by the timing of low-tide, sampling date and the time of day (Fig 3, Table 2). For example, irrespective of the percent cover of *P*. *perna*, body temperatures of both mussel species were greater (i) on days when the timing of low-tide was closer to midday, (ii) after low-tide compared to before, and (ii) on the 22 and 23 January 2015 when air temperatures were particularly high (Figs 2 and 3, Table 2). Body temperatures of individual mussels typically differed by 6°C to 8°C within a single 20 cm × 20 cm plot and at times by as much as 10.4°C and 11.8°C for *M*. *galloprovincialis* and *P*. *perna* respectively across the entire midshore region (Fig 3). Noticeably, this variability was highest during the hottest day of our sampling experiments, 23 January (Fig 3).

**Table 1:** Pearson correlations coefficients (r) for comparisons between the percent cover of *P*. *perna* and absolute/ relative temperatures in surface temperatures of *P*. *perna* and *M*. *galloprovincialis* measured one hour before and one hour after low-tide (LT) across four consecutive days in summer. Bold font highlights significant results.

	Absolute (*Perna*)			Absolute (*Mytilus*)			Relative temperature		
	r	n	p-value	r	n	p-value	r	n	p-value
**20 January 2015**									
1-hour before LT	0.062	29	0.190	0.001	26	0.867	0.086	26	0.145
1-hour after LT	0.075	29	0.150	0.012	28	0.583	0.157	28	**0.037**
**21 January 2015**									
1-hour before LT	0.035	29	0.328	0.001	28	0.892	0.314	28	**0.002**
1-hour after LT	0.004	29	0.758	0.001	27	0.995	0.017	27	0.512
**22 January 2015**									
1-hour before LT	0.010	29	0.601	0.001	27	0.927	0.317	27	**0.002**
1-hour after LT	0.035	29	0.330	0.061	28	0.206	0.009	28	0.626
**23 January 2015**									
1-hour before LT	0.007	27	0.686	0.001	25	0.969	0.037	25	0.356
1-hour after LT	0.037	28	0.758	0.001	24	0.968	0.014	24	0.585

**Table 2:** Three-way ANOVA of the effect of species, sampling date and time of day on shell surface temperatures of mussels during aerial exposure within the mid intertidal zone. Post-hoc comparisons represent the results of SNK tests. Bold font highlights significant results.

Source	df	MS	F-ratio	p-value
Species	1	7.8	5.73	**0.017**
Date	3	2091.3	1542.2	**<0.001**
Time	1	2541.1	1874.0	**<0.001**
Species*Date	3	0.5	0.35	0.231
Species*Time	1	2.0	1.44	0.786
Date*Time	3	298.3	220.01	**<0.001**
Species*Date*Time	3	0	0.02	0.995
Error	426	1.4		
Post-hoc (Species): *Perna > Mytilus*				
Post-hoc (Date*Time): 23^rd^(Before & After LT) >22^nd^(After LT)> 22^nd^(Before LT)>21^st^(After LT)> 21^st^(Before LT)>20^th^(Before & After LT)				

### Density manipulations

**Fig 4:**
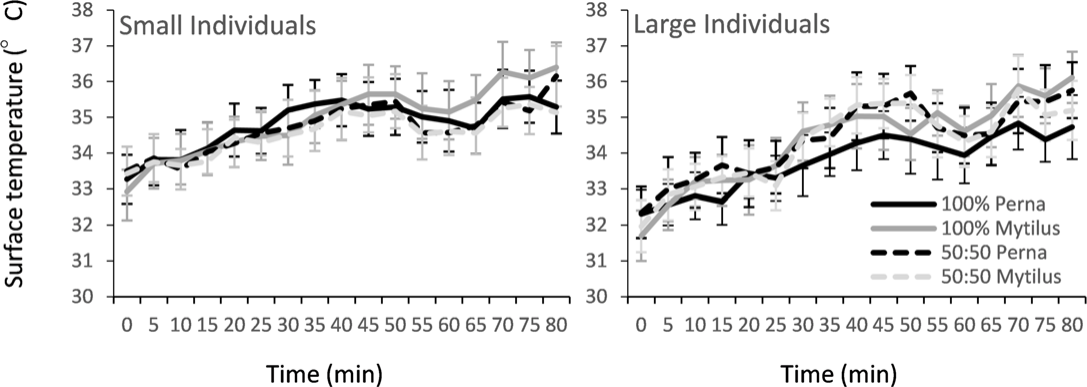
Changes in mean surface temperatures of *P*. *perna* and *M*. *galloprovincialis* amongst mixed and monspecific artificial mussel beds after being emersed from water (i.e. time zero) (n = 3 plots). Left figure represents mussel beds comprised of small individuals (4 to 5.5 cm shell length), right figure large individuals (6 to 7.5 cm).

Mean body temperatures of *M*. *galloprovincialis* within monospecific beds reached higher temperatures than *P*. *perna* placed within monospecific beds (Fig 4, Table 3). This was true irrespective of whether beds were comprised of small or large individuals (Table 3). Body temperatures of both *M*. *galloprovincialis* and *P*. *perna* were similar within mixed mussel beds and after 80 min of exposure body temperatures of *M*. *galloprovincialis* within mixed mussel beds were significantly cooler than their monospecific counterparts, and *vice versa* for *P*. *perna* within mixed mussel beds (Table 3). Body temperatures of mussels increased significantly faster within 100% *M*. *galloprovincialis* and mixed mussel beds compared to 100% *P*. *perna* (Table 3). Body temperatures also increased faster within beds comprised of large as opposed to small individuals (Table 3). However, there was no interaction between density treatment and size of individuals within artificial mussel beds (Table 3).

**Table 3:** Two-way ANOVA of the effect of density manipulations on (i) body temperatures of *P*. *perna* and *M*. *galloprovincialis* 40 min and 80 min after exposure to aerial heat-stress, and (ii) body heating rates (degrees per min) during the first 90 min of experiment. Post-hoc comparisons represent the results of SNK tests. Bold font highlights significant results.

Source	df	MS	F-ratio	p-value
***Body temperature***				
*40 min*				
Density	3	0.199	0.625	0.609
Size	1	0.616	1.933	0.184
Density*Size	3	0.573	1.797	0.188
Error	16	0.320		
*80 min*				
Density	3	2.178	3.842	**0.030**
Size	1	0.520	0.918	0.352
Density*Size	3	0.102	0.180	0.908
Error	16	0.570		
Post-hoc: 100% *Mytilus* > Mixed (*Mytilus*) = Mixed (*Perna*) > 100% *Perna*				
***Body heating rate***				
Density	3	0.001	4.66	**0.016**
Size	1	0.001	6.51	**0.021**
Density*Size	3	0.001	0.44	0.727
Error	16	0.001		
Post-hoc (Density): 100% *Mytilus*, Mixed (*Perna*) & Mixed (*Mytilus*) > 100% *Perna*				
Post-hoc (Size): Large > Small individuals				

**Fig 5:**
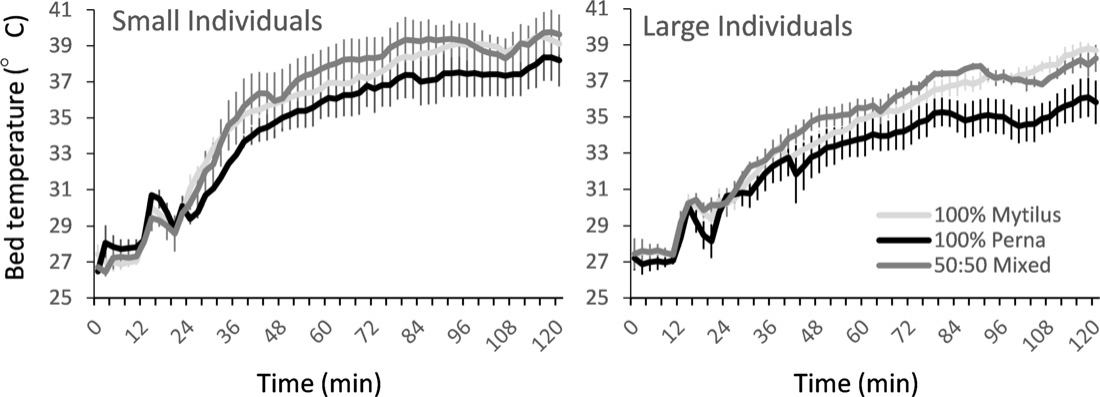
Changes in mean bed temperatures in mixed and monspecific artificial mussel beds after being emersed from water (i.e. time zero) (n = 3 loggers). Left figure represents mussel beds comprised of small individuals (4 to 5.5 cm shell length), right figure, large individuals (6 to 7.5 cm).

As for body temperature, differences in bed temperatures and heating rates were largely dependent on the size of mussels within the bed (Fig 5, Table 4). In contrast to body temperatures, however, bed temperatures and heating rates were greater amongst mussel beds comprised of small individuals. Whilst not statistically significant, bed temperatures increased faster and were somewhat greater amongst cages filled with either 50% or 100% *M*. *galloprovincialis* than mussel beds comprised of 100% *P*. *perna*, the difference increasing with time (Fig 5).

**Table 4:** Two-way ANOVA on the effect of the density manipulations on (i) bed temperature 60 min and 120 min after exposure to aerial heat-stress, and (ii) habitat heating rate (degrees per min) during the first 90 min of the experiment. Post-hoc comparisons represent the results of SNK tests. Bold font highlights significant results.

Source	df	MS	F-ratio	p-value
***Bed temperature***				
*60 min*				
Density	2	4.46	2.11	0.165
Size	1	22.67	10.70	**0.007**
Density*Size	2	0.05	0.03	0.975
Error	12	2.12		
Post-hoc: Small > Large individuals				
*120 min*				
Density	2	6.33	2.086	0.167
Size	1	11.49	3.789	0.075
Density*Size	2	1.62	0.534	0.599
Error	12	3.03		
***Bed heating rate***				
Density	2	0.001	2.72	0.106
Size	1	0.002	6.17	**0.029**
Density*Size	2	0.001	0.01	0.996
Error	12	0.001		
Post-hoc: Small > Large individuals				

## Discussion

Artificially manipulating the densities of two competing intertidal mussel species significantly reduced the capacity of the indigenous species *P*. *perna* for group thermoregulation during periods of heat-stress. Conversely, within mixed mussel beds, the invasive mussel *M*. *galloprovincialis* appeared to benefit from the evaporative cooling behaviour employed by *P*. *perna* since body temperatures of *M*. *galloprovincialis* in mixed beds remained significantly cooler than conspecifics in monospecific beds. The results also suggest that irrespective of species or thermoregulatory behaviour, mussel beds comprised of larger individuals are more effective at reducing thermal stress across the entire mussel bed. This supports current physiological principles that suggest the greater body mass of large individuals will act as a buffer against changes in abiotic conditions and help maintain relatively stable body temperatures [2]. However, it should be noted that the size of individual mussels within beds only affected bed temperature and not the body temperatures of individual mussels and that heating rates of body temperatures increased with an increase in the size of individual mussels within a bed. The cause of this discrepancy between mussel bed and mussel body temperatures is unknown.

The thermal relationship between the two species also supports previous laboratory and field-based studies showing that mussels within monospecific beds of *P*. *perna* are able to maintain lower body temperatures and show better survival than mussels within monospecific *M*. *galloprovincialis* beds [23]. The fact that *M*. *galloprovincialis* 'steals' the cooling effect produced by gaping *P*. *perna* may contribute to the ecological success of *M*. *galloprovincialis*, especially along the southern coast of South Africa. More importantly, however, it indicates how the response of a species to changes in ambient temperature can be shaped by indirect interactions with other species.

Unlike artificial beds, body temperatures of *M*. *galloprovincialis* and *P*. *perna* in natural mussel beds within the midshore region did not vary as a function of species composition. Instead, body temperatures of both species appeared to vary depending on the timing of low-tide, the time of day and differences in maximum air temperatures, which supports previous findings [29, 30]. Temperature variation amongst plots, and individuals within plots, was also high and comparable to that found in other intertidal studies [31–33]. Differences in the effect of species composition between natural and artificial beds could be related to the fact that the size and structure of natural mussel beds is inherently more variable (e.g. mixed size classes, multi-layered) and distributed across a wider range of topographies than the artificial beds used in this study. Additionally, mussels in natural beds were often covered with encrusting flora and fauna whilst those in artificial beds were not. Thus, variability associated with small-scale differences in topography, size-structure, mussel bed size and the presence of encrusting organisms could have masked the effects of thermoregulatory behaviour within natural mussel beds.

Increasing temperatures associated with climate change are expected to favour positive biological interactions [34]. However, as average global temperatures continue to rise, we might expect the negative effects of *M*. *galloprovincialis* on *P*. *perna* to become stronger. For example, many intertidal communities are expected to undergo further ‘coastal squeeze’, which results from rising sea-levels forcing species higher up the shore and higher atmospheric temperatures and heat-stress forcing species further down, increasing competition for primary substrata [35]. Consequently, due to reductions in the availability of primary substrata alone, we might expect increased levels of competition between *P*. *perna* and *M*. *galloprovincialis*, thus theoretically reducing the ability of *P*. *perna* to thermoregulate. This might not initially have negative impacts on populations of *P*. *perna* since small increases in body temperatures of ectothermic species may increase metabolism, growth and reproduction. However, if temperatures surpass a physiological threshold the benefits of increased body temperatures will soon become detrimental.

## Conclusion

The results of our study highlight the complexity of interspecific interactions and how these can influence thermoregulation of one or more species. We show that small-scale changes in the relative abundances of an intertidal ectotherm can influence the thermal characteristics of another ectotherm through differences in thermoregulatory behaviour. This finding highlights the fact that the responses of species to changing environmental conditions need to be viewed within the context of both direct and indirect biological interactions.

## Supporting Information

S1 Fig Size frequency distributions of the indigenous mussel (*Perna perna*) and the invasive mussel (*Mytilus galloprovincialis*) at the Jongensfontein, South Africa. (DOCX)
