# Supplementary material for: Cheating the Locals: Invasive Mussels Steal and Benefit from the Cooling Effect of Indigenous Mussels
Source: PLoS One. 2016 Mar 31;11(3):e0152556. doi: 10.1371/journal.pone.0152556 (PMC4816446; doi:10.1371/journal.pone.0152556)
Supplement: S1 Fig — (DOCX) [file pone.0152556.s001.docx]

**Supporting information**

***S1 Text. Size frequency distributions of the indigenous mussel (Perna perna) and the invasive mussel (Mytilus galloprovincialis) at the Jongensfontein, South Africa***

Frequency (%)

Frequency (%)

Size class (mm)

**Fig S1.** Size-frequency distributions for maximum shell length of *Perna perna* (n=1022) and *Mytilus galloprovincialis* (n=476) at Jongensfontein in January 2015.
